# CkP1 bacteriophage, a S16-like myovirus that recognizes *Citrobacter koseri* lipopolysaccharide through its long tail fibers

**DOI:** 10.1007/s00253-023-12547-8

**Published:** 2023-05-03

**Authors:** Hugo Oliveira, Sílvio Santos, Diana P. Pires, Dimitri Boeckaerts, Graça Pinto, Rita Domingues, Jennifer Otero, Yves Briers, Rob Lavigne, Mathias Schmelcher, Andreas Dötsch, Joana Azeredo

**Affiliations:** 1 Centre of Biological Engineering, University of Minho, Braga, Portugal; 2 LABBELS –Associate Laboratory, Braga, Guimarães Portugal; 3 Department of Biotechnology, Ghent University, Ghent, Belgium; 4 Departament de Genètica I de Microbiologia, Universitat Autònoma de Barcelona, Barcelona, Spain; 5 Department of Biosystems, KU Leuven, Louvain, Belgium; 6 Institute of Food, Nutrition and Health, ETH Zurich, Zurich, Switzerland; 7 Max Rubner-Institute, Department of Physiology and Biochemistry of Nutrition, Karlsruhe, Germany

**Keywords:** *Citrobacter*, Bacterial infection, Bacteriophage, Long tail fiber, Control, Diagnostics

## Abstract

**Abstract:**

*Citrobacter koseri* is an emerging Gram-negative bacterial pathogen, which causes urinary tract infections. We isolated and characterized a novel S16-like myovirus CKP1 (vB_CkoM_CkP1), infecting *C. koseri*. CkP1 has a host range covering the whole *C. koseri* species, *i.e.*, all strains that were tested, but does not infect other species. Its linear 168,463-bp genome contains 291 coding sequences, sharing sequence similarity with the *Salmonella* phage S16. Based on surface plasmon resonance and recombinant green florescence protein fusions, the tail fiber (gp267) was shown to decorate *C. koseri* cells, binding with a nanomolar affinity, without the need of accessory proteins. Both phage and the tail fiber specifically bind to bacterial cells by the lipopolysaccharide polymer. We further demonstrate that CkP1 is highly stable towards different environmental conditions of pH and temperatures and is able to control *C. koseri* cells in urine samples. Altogether, CkP1 features optimal in vitro characteristics to be used both as a control and detection agent towards drug-resistant *C. koseri* infections.

**Key points:**

•* CkP1 infects all C. koseri strains tested*

•* CkP1 recognizes C. koseri lipopolysaccharide through its long tail fiber*

•* Both phage CkP1 and its tail fiber can be used to treat or detect C. koseri pathogens*

**Supplementary Information:**

The online version contains supplementary material available at 10.1007/s00253-023-12547-8.

## Introduction

*Citrobacter* species are Gram-negative bacilli that belong to the *Enterobacteriaceae* family. The genus is composed of about a dozen species, but only *Citrobacter freundii*, *Citrobacter koseri* (formerly named *Citrobacter diversus*), and *Citrobacter amalonaticus* are pathogenic to humans. In particular, *C. koseri* accounts for a variable, yet large, portion of *Citrobacter* infections (ranging from 20 to 90%), often associated with urinary tract infections (Ranjan and Ranjan 2013; Deveci and Coban 2014). *C. koseri* strains are naturally resistant to ampicillin but can rapidly gain resistance to other antibiotics (*e.g.*, ciprofloxacin, cefuroxime, aztreonam, ceftazidime, gentamicin) by chromosomal or mobile genetic determinant elements, thus creating a need for alternative therapies (Rizvi et al. 2010; Aruna and Mobashshera 2012).

Bacteriophages (phages) have emerged as a possible solution to control drug-resistant pathogens linked to human infectious diseases (Melo et al. 2020). Broad host range phages can be more advantageous to control pathogens compared to narrow-host-range phages, avoiding the need to identify the causative agent prior to treatment. Phages of the recently defined *Straboviridae* family and *Tevenvirinae* (T-even) subfamily, which have a myovirus morphotype, have been found as a good source of broad-spectrum viral candidates targeting different bacterial pathogens, at both species and genus levels. For instance, phage T4 is reported to have a relatively broad host range within the *Escherichia coli* species, whereas phage S16 exhibits broad host range within the *Salmonella* genus (Marti et al. 2013). There are also other T-even broad-host-range reported against *Salmonella*, for instance, phage STP4-a (Li et al. 2020) and phage SHWT1 (Tao et al. 2021).

A common feature to all T-even phages is that their host interaction is mediated by an initial, reversible binding with the long tail fiber (LTF), followed by a second and irreversible binding with the short tail fibers. The reversible binding defines the phage host range. In phages T4, T2, and S16, gp34 to gp38 are involved in the production of LTF proteins, from proximal to distal segments (King and Laemmli 1971; Riede et al. 1985; Riede 1987; Dunne et al. 2018). While in T4, gp38 functions as a chaperone (Hashemolhosseini et al. 1996), in T2 and S16, gp38 acts as an adhesin that attaches to the mature LTF and modulates the receptor specificity (Riede et al. 1985, 1987; Marti et al. 2013).

Recently, more T-even phages have been isolated and characterized (*e.g.*, R3, EC14, EC35, and 8S coliphages). High-throughput techniques have confirmed that they use lipopolysaccharide (LPS), LamB, OmpA, and OmpC as surface receptors (Kortright et al. 2020). Similarly, *Yersinia pestis* myoviruses with equivalent properties, *i.e.*, broad host range, use their long tail fiber tips for Omp and LPS recognition (Chen et al. 2020; Li et al. 2020; Salem et al. 2021).

To broaden the scope of phage therapy against emerging *Citrobacter* infections, here we report the isolation of a new T-even *C. koseri*-infecting phage vB_CkoM_CkP1 (further mentioned as CkP1). CkP1 has the typical genomic, morphologic, and infection/stability features of other T-even phages. We show that CkP1 has a host range covering all strains tested within *C. koseri* species and that it recognizes the LPS using the LTF (gp267) with high nanomolar equilibrium affinity. Furthermore, we predict protein structures of gp267 using AlphaFold-Multimer and compare to known tail fiber structures of S16 and T4 (Jumper et al. 2021; Evans et al. 2022). Finally, we demonstrate that CkP1 is efficient in controlling *C. koseri* in urine. Altogether, CkP1 displays optimal traits that can be used to detect and control *C. koseri* infections.

## Materials and methods

### Bacterial strains

Sixty strains were used in this study, including the collection and clinical isolates from the Hospital de Braga (Braga, Portugal) collected over a period of 3 years, and strains kindly provided by Tomáš Kuchta lab in Slovakia (CK#6, CK#9, CF#7, CB) or from the Salmonella Genetic Stock Centre (CK#22, CK#25, CF#1, CA#1, MM#1, SE#1), Spanish Type Culture Collection (PV, EC), and American Type Culture Collections (KP, SE#2) or standard laboratory *E. coli* strains (Top10, K12) (Table 1 and associated footnote). The panel includes several *Citrobacter* species, *i.e.*, *C. koseri* (*n* = 25), *C. freundii* (*n* = 21), *C. amalonaticus* (*n* = 1), *C. youngae* (*n* = 1), and *C. braakii* (*n* = 1), as well as representatives of other closely related species. For the clinical isolates of the Hospital de Braga, human samples were first cultured in agar plates, including blood agar, chocolate blood agar, CLED agar, or MacConkey agar, from which *Citrobacter* strains were isolated after 18 to 24 h of incubation at 35 °C. Typing was performed using MALDI-TOF MS or Gram-negative identification cards of Vitek2 (bioMérieux) or WalkAway (Beckaman Coulter). Next, susceptibility profiles were performed using antibiogram cards for Vitek2 and WalkAway, covering imipenem, meropenem, and ertapenem, according to EUCAST guidelines. Beta-lactamases (AmpC) and extended-spectrum beta-lactamase (ESBL)-producing bacteria were identified using an Etest (bioMérieux) with positive results for cefotetan/cefotetan + cloxacillin and for cefepime/cefepime + clavulanic acid, respectively. All strains were grown at 37 °C in Tryptic Soy Broth (TSB, VWR) with or without agar (Merck). Different agar concentrations of 1.2% or 0.6% were used to prepare Tryptic soy agar plates (TSA) and soft agar overlays, respectively.

**Table 1:** Lytic spectra and efficiency of plating of the *Citrobacter* phage CkP1

CkP1 phage							
Species	Strain^1^	Origin	Isolation date (dd/mm/yyyy)	Patient gender	Antibiotic resistances^2^	Infectivity	EOP^3^
*C. koseri*	CK#1	Pus	28/04/2014	Male	AM	+	(1.0) High
	CK#2	Urine	05/05/2014	Male	AM, AMC, CXM	+	(0.7) High
	CK#3	Skin	24/04/2014	Female	AM, CXM	+	(0.8) High
	CK#4	Urine	22/04/2014	Female	AM	+	(0.7) High
	CK#5	Biopsy Mat	28/04/2014	Male	AM	+	(0.9) High
	CK#6	Unknown	Unknown	Unknown	Unknown	+	(0.8) High
	CK#7	Urine	27/05/2014	Male	AM, CXM	+	(0.9) High
	CK#8	Urine	28/05/2014	Male	AM	+	(1.1) High
	CK#9	Unknown	Unknown	Unknown	Unknown	+	(0.5) High
	CK#10	Urine	22/09/2014	Female	AM	+	(0.8) High
	CK#11	Urine	08/11/2014	Male	AM	+	(0.6) High
	CK#12	Ocular excess	27/01/2017	Male	AM	+	(0.7) High
	CK#13	Urine	05/11/2014	Male	AM	+	(0.8) High
	CK#14	Urine	24/10/2014	Male	AM, AMC, GM, NN, TZP, SXT	+	(0.8) High
	CK#15	Bronchial aspirate	07/11/2014	Male	AM	+	(1.0) High
	CK#16	Urine	06/02/2017	Female	AM	+	(1.1) High
	CK#17	Urine	06/02/2017	Male	AM, CXM, TZP	+	(0.9) High
	CK#18	Urine	28/08/2017	Female	AM	+	(0.6) High
	CK#19	sputum	03/05/2017	Female	AM	+	(0.9) High
	CK#20	skin	28/04/2017	Female	AM, CXM	+	(0.6) High
	CK#21	Urine	19/04/2017	Female	AM	+	(0.5) High
	CK#22^1^	Unknown	Unknown	Unknown	Unknown	+	(0.9) High
	CK#23	Urine	20/10/2017	Female	AM	+	(0.7) High
	CK#24	Urine	29/10/2017	Male	AM	+	(0.8) High
	CK#25^1^	Unknown	Unknown	Unknown	Unknown	+	(1.0) High
*C. freundii*	CF#1^1^	Unknown	Unknown	Unknown	Unknown	−	
	CF#2	Feces	30/04/2014	Female	Unknown	−	
	CF#3	Feces	03/05/2014	Female	Unknown	−	
	CF#4	Urine	15/04/2014	Male	AM, CXM, SXT	−	
	CF#5	Urine	24/05/2014	Female	AM, AMC, CXM, SXT, MEM, TZP, CT, TZ, CIP, GM, NN, AN, ESBL, AmpC	−	
	CF#6	Feces	30/05/2014	Male	Unknown	−	
	CF#7	Unknown	Unknown	Unknown	Unknown	−	
	CF#8	Feces	05/05/2014	Female	Unknown	−	
	CF#9	Urine	12/06/2014	Female	AM, AMC, CXM, SXT, MEM, TZP, CT, TZ, CIP, GM, NN, AN, ESBL, AmpC	−	
	CF#10	Urine	12/06/2014	Female	AM, AMC, CT, TZ, CXM, TZP	−	
	CF#11	Urine	11/06/2014	Male	AM, AMC	−	
	CF#12	Feces	12/05/2014	Female	Unknown	−	
	CF#13	Urine	14/05/2014	Female	AM, AMC, CXM	−	
	CF#14	Urine	13/05/2014	Female	Unknown	−	
	CF#15	Peritoneal fluid	14/05/2014	Female	Unknown	−	
	CF#16	Urine	07/05/2014	Male	AM, AMC	−	
	CF#17	Pus	05/05/2014	Male	AM, AMC	−	
	CF#18	Urine	11/05/2014	Male	AM, AMC	−	
	CF#19	Urine	15/04/2014	Female	AM, AMC	−	
	CF#20	Urine	04/11/2014	Male	AM, AMC	−	
	CF#21	Urine	05/11/2014	Male	AM, AMC	−	
*C. amalonaticus*	CA#1^1^	Unknown	Unknown	Unknown	Unknown	−	
	CA#2	Peritoneal fluid	14/05/2014	Female	AM, CXM	−	
*C. youngae*	CY	Feces	08/05/2014	Male	Unknown	−	
*C. braakii*	CB	Unknown	Unknown	Unknown	Unknown	−	
*M. morganii*	MM^1^	Urine	Unknown	Female	Unknown	−	
*P. stuartii*	PS	Urine	Unknown	Female	AM, AMC, CXM	−	
*P. rettgeri*	PR		Unknown	Unknown	Unknown	−	
*P. mirabilis*	PM^1^	Unknown	Unknown	Unknown	Unknown	−	
*P. vulgaris*	PV^1^	Unknown	Unknown	Unknown	Unknown	−	
*E. coli*	EC#1^1^	Unknown	Unknown	Unknown	Unknown	−	
	EC#2^1^	Unknown	Unknown	Unknown	Unknown	−	
*K. pneumoniae*	KP^1^	Unknown	Unknown	Unknown	Unknown	−	
*S. enterica*	SE#1^1^		Unknown	Unknown	Unknown	−	
	SE#2^1^		Unknown	Unknown	Unknown	−	

^1^Collection strains: CK#22-SGSC 5610; CK#25-SGSC 4696; CF#1-SGSC 5345; CA#1-SA 5615; MM#1-SGSC 5703; PV-CECT 174; EC-CECT 432; KP-ATCC 11,296, SE#1-SGSC 3029, SE#2-ATCC 13,076; reference strains: EC#1-Top10, EC#2-BL21

^2^Antibiotic resistances: *AM* ampicillin, *AMC* amoxicillin/clavulanic acid, *CXM* cefuroxime, *SXT* cotrimoxazole, *TZP* piperacillin/tazobactam, *MEM* meropenem, *CT* cefotaxime, *TZ* ceftazidime, *CIP* ciprofloxacin, *GM* gentamicin, *NN* tobramycin, *AN* amikacin, *ESBL* extended-spectrum beta-lactamase producing bacteria, *AmpC* beta-lactamase producing bacteria

^3^The relative efficiency of plating was calculated by dividing each phage titer (PFU/ml) by the phage titer of the propagating host (CK#1) and recorded as high (≥ 0.5) or low (< 0.5)

### Phage isolation

Phage CkP1 was isolated from sewage water from an urban wastewater treatment plant at Frossos (Braga, Portugal) using the enrichment procedure, production and purification, exactly as described previously (Oliveira et al. 2016a, b). Phage stocks were stored in SM buffer (50 mM Tris–HCl pH 7.5, 100 mM NaCl, 8 mM MgSO_4_) at 4 °C.

### Lytic spectra and efficiency of plating

The phage CkP1 host range and efficiency of plating (EOP) was established against all strains listed in Table 1. Bacterial lawns were prepared on TSA plates by adding 100 μl of exponential-phase cell culture of each strain, and subsequent spotting of 10 μl of phage (10^8^ PFU/ml and ten-fold dilution series). After a 16-h incubation period at 37 °C, results were scored. The relative EOP was calculated by dividing the titer of the phage (PFU/ml) for each isolate by the titer for the propagating host (CK#1) and recorded as high (≥ 0.5) or low (< 0.5).

### One-step growth curve

One-step growth curve experiments were performed as previously described (Sillankorva et al. 2008). Briefly, 10 ml of a mid-exponential-phase *C. koseri* CK#1 cells (OD_620_ = 0.5) was harvested by centrifugation (7000 × *g*, 5 min, 4 °C) and resuspended in 5 ml fresh TSB medium. Next, phage CkP1 was added to the prepared host suspensions with a multiplicity of infection (MOI) of 0.001 (in a final volume of 10 ml). Phages were allowed to adsorb for 5 min at 37 °C, 120 rpm (Biosan ES-20/60). The mixtures were subsequently centrifuged (7000 × *g*, 5 min, 4 °C) and the pellets were resuspended in 10 ml of fresh TSB medium. Samples were taken every 5 min over a period of 30 min and then every 10 min until 1 h of infection. The phage concentration was assessed by plating 100 μl of tenfold serial dilutions, previously mixed with 100 μl of the overnight cultured host and 3 ml of soft-agar TSA (0.6% agar). Averages ± standard deviations for all experiments are shown for *n* = 3 repeats.

### Phage stability

Tests were performed to evaluate the phage CkP1 stability at either different temperature or pH values, as previously described (Oliveira et al. 2016a, b). Thermal stability was established by incubating 10^8^ PFU/ml of the phage at − 20, 4, 37, 42, 50, and 60 °C for 24 h. Similarly, the effect of pH was also evaluated for pH values of 1, 3, 5, 7, 9, 11, and 13 using a universal pH buffer (150 mM potassium chloride, 10 mM potassium dihydrogen phosphate, 10 mM sodium citrate, 10 mM boric acid). In both experiments, phages were diluted and plated on *C. koseri* CK#1 lawns for enumeration. Averages ± standard deviations for all experiments are indicated for *n* = 3 repeats.

### Electron microscopy

Phage particles were examined with a Jeol JEM 1400 transmission electron microscope (TEM), exactly as described before (Oliveira et al. 2017).

### Phage DNA isolation, sequencing, and annotation

Phage DNA was extracted from highly concentrated and purified phage stock (≥ 10^10^ PFUs/ml) using the standard procedures described elsewhere (Sambrook and Maniatis 1989). A 2 × 150 bp paired-end DNA library (Nextera XT sample prep) was prepared and sequenced with an Illumina MiSeq platform (Illumina Inc., USA) at the Nucleomics Core (VIB, Belgium). After processing, reads were trimmed to remove adapters, contaminations, or low-quality nucleotides and then de novo assembled with CLC Genomics Workbench version 7.0 (CLC Bio, Aarhus, Denmark). PCR amplifications and Sanger sequencing were performed to verify regions displaying uncertain consensus sequence. In addition, contigs were scaffolded by amplification and Sanger sequencing (Eurofins Genomics) of fragments that covered the remaining gaps.

The genome was annotated using myRAST (Aziz et al. 2008) and manually inspected. Sequence similarity detection and secondary structure prediction was performed using BLASTp (Altschul et al. 1990) and HHPRED (Soding et al. 2005), respectively. tRNAs were predicted using tRNAscan-SE (Schattner et al. 2005). The DNA homology comparisons between phage genomes were performed with BLASTN and visualized with Easyfig (Sullivan et al. 2011). The shared protein content was conducted with Orthovenn2 (Xu et al. 2019).

### Tail fiber cloning, expression, and purification

The LTF protein (gp267) was fused to the *Aequorea coerulescens* green florescence protein (GFP) and produced recombinantly after co-expression with and without the putative chaperone (gp268) as a bicistronic transcript in pET28a. Three open reading frames were made: GFP-gp267 (GFP fused with the gp267 LTF); GFP-gp267/gp268 (GFP fused with the gp267 and co-expressed with the putative chaperone gp268); and GFP-gp267trunc/gp268 (GFP fused with a truncated variant of gp267 (starting at nucleotide 1249, *i.e.*, amino acid residue 417, designed to use the predicted C-terminal binding domain only and co-expressed with gp268). Genes *267* and *268* were amplified with Phusion™ High-Fidelity DNA Polymerase (Thermo Fisher Scientific) using phage CkP1 genomic DNA as template with the primers Fw 5′-CCGCCGGGATCCGAATTCATGGCAACTATTAAGCAAATACAATTAAAAAGAAG, Fw 5′-CGCCGGGATCCGAATTCATGTGGGGAACTGGTGGTTTAAAAG, Rv 5′-GCCGCCGTCGACTTAACCGATTCTTACAATATATTTAAACGCTAC, and Rv 5′-CCGCCGCTCGAGTTAGCCATGTGAGTGTCTATGGAATAG, containing the BamHI/SalI/XhoI restriction sites (underlined). Amplicons were cleaned (DNA Clean & Concentrator-5 k, Zymo Research, USA), digested with the listed fast digest restriction enzymes (Thermo Fisher Scientific) and ligated with a T4 ligase (Thermo Fisher Scientific) into the previously constructed pET28a with GFP (Santos et al. 2019). All constructs contain an N-terminal 6xHis-tag followed by the *gfp* upstream the phage gene(s). The different constructs were used to transform *E. coli* TOP10 competent cells (Invitrogen). Colonies were screened through colony PCR and the positive ones were used for plasmid extraction and further confirmation through Sanger sequencing (Eurofins Genomics). The pET28a plasmid with GFP was used as control in cloning and expression. Correct plasmids were used to transform competent *E. coli* Arctic Express cells (DE3) (Agilent) to express the proteins when reaching a mid-exponential phase (optical density at 620 nm of 0.6), with 1 mM isopropyl β-d-1-thiogalactopyranoside (Sigma-Aldrich), 24 h at 10 °C, 180 rpm. Protein purification was performed on an immobilized metal affinity chromatography column using an imidazole gradient (25 to 250 mM). Next, samples were dialyzed against in 10 mM Tris–HCl (pH = 7) buffer and quantified with the Pierce BCA Protein Assay Kit (Thermo Fisher) as described previously (Oliveira et al. 2016a, b).

### Tail fiber binding

#### Fuorescence microscopy

The binding ability of the different gp267 LTF constructs was inferred by fluorescence microscopy observations of the *C. koseri* cells after incubation with each three fusion proteins. GFP alone was used as negative control. Briefly, bacterial cells were grown in TSB medium at 37 °C to the mid-log phase (OD_620nm_ = 0.3–0.4) and washed with 10 mM Tris–HCl (pH = 7). A pellet from a 100-µl suspension was resuspended in 20 µL of 10 µM purified gp267 LTF and incubated for 15 min at 20 °C. Cells were washed twice in 10 mM Tris–HCl to remove the unbound protein. The washed pellet was suspended in 10 µl of 10 mM Tris–HCl (pH = 7) and observed under an epifluorescence microscope equipped with U-RFL-T light source (Olympus BX51, Magnitude 1000 ×) in bright field and under the FITC (470–490, LP-516) filter. Control samples using 10 mM Tris–HCl (pH 7) or GFP alone (instead of the fused recombinant gp267 LTF) were prepared simultaneously.

#### Binding kinetics of tail fiber constructs to *C. koseri* cells

Binding kinetics and affinity of the full-length and truncated C-terminal version of the GFP-LTF constructs to the surface of *C. koseri* cells were determined by surface plasmon resonance (SPR) analysis, using a BIAcore X system (GE Healthcare Life Sciences) and C1 sensor chips, essentially as previously described (Schmelcher et al. 2010). In brief, the GFP-gp267trunc/gp268 protein was immobilized on the sensor chip surfaces in both flow cells (Fc1 and Fc2) using the amine coupling procedure (70 µl of 0.5 mg/ml at a flow rate of 5 µl/min). Then, inactivated *C. koseri* cells (4% of paraformaldehyde fixed) in HBS-T running buffer (10 mM HEPES–NaOH, 150 mM NaCl, 3.4 mM EDTA, 0.005% Tween 20, pH 7.8) were immobilized onto the protein lawn in Fc2 (15 µl of ~ 2 × 10^9^ CFU/ml at a flow rate of 3 µl/min). Finally, real-time interactions between the immobilized cells in Fc2 and the proteins of interest (GFP-gp267, GFP-gp267/gp268, and GFP-gp267trunc/gp268) were measured (30 µl at a flow rate of 10 µl/min) at different concentrations (1 nM to 1 µM). The Fc1 cell served as a reference in this experiment. Association phases were recorded for 3 min and dissociation phases for 12 min. Between measurements of different concentrations of the same protein, the chip surface was regenerated by one to three short pulses (10 to 20 µl) of regeneration buffer (10 mM HEPES–NaOH, 1 to 2 M NaCl, pH 7.8). Kinetic data were evaluated by global fittings with the “1:1 binding with mass transfer” and “two-state reaction” models predefined in the BIAcore evaluation software.

### Structure prediction with AlphaFold-Multimer

The trimeric structures of the N-terminal (AA 1–416) and C-terminal part (AAs 417–670) of the gp267 protein were predicted using AlphaFold-Multimer (v2.2.2) that was deployed on Ghent University’s supercomputing infrastructure (Jumper et al. 2021; Evans et al. 2022). Two NVIDIA Ampere A100 graphics processing units (GPUs) with 80 GB of GPU memory were used. Predicted structures were inspected for their accuracy by computing and visualizing the predicted local distance difference test (pLDDT) scores, the predicted template modeling scores, and alignment errors, using code from ColabFold (Mirdita et al. 2022). Both predicted structures were subjected to FoldSeek, a search and comparison tool for protein structures, to detect and compare our predictions to other known structures (van Kempen et al. 2022).

### Phage receptor

#### Generation of phage-resistant variants

Two different approaches were used to identify the phage receptor. First, *C. koseri* SGSC4696 (GenBank accession no. NC_009792) strain mutants were generated using the Datsenko technique (Datsenko and Wanner 2000). Recombinogenic plasmid pMJH46 (Addgene) was transferred to electrocompetent *C. koseri* SGSC4696 cells. Next, homologous sequences were chosen to delete either *ompC* (CKO_RS02455) (Fw 5′-ATGTTACGCAGCAGCAAC; Rv 5′-TTAGGTGGCGGTACTTGG) or *ompA* (CKO_RS08945) (Fw 5′-ATGTTACGCAGCAGCAAC; Rv 5′-TTAGGTGGCGGTACTTGG) genes or both combined, using the listed primers with 50-bp recombination arms. Deletion mutants were confirmed by Sanger sequencing (Eurofins Genomics). Secondly, five spontaneous *C. koseri* SGSC4696 variants displaying resistances to the phage CkP1 after incubation overnight on TSA plaques were selected for genome sequencing. DNA was extracted from overnight cultures using the Quick-DNA Fungal/Bacterial Miniprep Kit (Zymo Research). To prepare DNA fragments (DNA libraries), 1 µg of genomic DNA was fragmented using the Bioruptor sonicator (Diagenode, Denville, NJ) and then prepared using the KAPA HyperPrep kit (KAPA Biosystems, Wilmington, MA) according to the supplier’s protocol. The DNA libraries were sequenced in the lllumina NovaSeq platform, using 150-bp paired-end sequencing reads (Stabvida). The samples generated from 1122 Mbp (7,435,112 sequence reads) to 1465 Mbp (9,704,010 sequence reads) were assembled, resulting in a theoretical average coverage ranging from 238 × to 311x (assuming a genome size of 4.7 Mbp).

To detect small genomic variations (single nucleotide variants, SNVs, and insertions/deletions (indels)), the genomic data obtained for the variants were mapped to the reference genome of the *C. koseri* wild type (GenBank accession no. NC_009792) using *bowtie2* (v2.3.3.1) with option --*local* (Langmead and Salzberg 2012), resulting in an overall alignment rate of > 96% for each variant. Candidate small variations were detected using *samtools* (v1.9) (Li et al. 2009; Li 2011) with a custom shell script (code accessible upon request), filtering for variants covered by at least five reads, and confirmed by visually inspecting the mapped sequence data in the *Integrative Genomics Viewer* (*IGV*) (Robinson et al. 2017). Larger deletions were searched by identification of regions with low coverage (< 10 reads) using *bedtools* (v2.26.0) (Quinlan 2016) but no deletions were identified after visual inspection of these regions in IGV. As a complementary approach, the variant genomes were also assembled de novo using *SPAdes* (v3.13.1) (Bankevich et al. 2012) using default parameters and the reference genome NC_009792 as “trusted contig.” The resulting contigs were aligned with the reference genome using *progressiveMauve* (Darling et al. 2010) to identify potential large genomic variations or rearrangements.

#### Complementation strain construction

The *rfaF* (CKO_RS21630) and the *gt* (CKO_RS21655) genes were amplified by PCR with Phusion High-Fidelity DNA Polymerase (Thermo Scientific) using the *C. koseri* SGSC4696 (accession no. NC_009792) genomic DNA as template and using the primers Fw 5′-CCATGGCCATGAAAATACTGGTGATTGGTC, Rv 5′-AAGCTTTCAGGCTTCCTCTTGTAATAA and Fw 5′-CCATGGCCATGGATAACAAAACTAACGTAATTAATATTGC, and Rv 5′-AAGCTTTTAATGAATCTTGGTTTTGAGGTACTTT, respectively, containing the NcoI/HindIII restriction sites (underlined). The amplicons were digested with the FastDigest NcoI/HindIII restriction enzymes (Thermo Scientific), ligated into pET28a with a T4 DNA ligase (Thermo Scientific) according to the manufacturer’s instructions, and used for transformation of *E. coli* TOP10 electrocompetent cells (25 μF, 200 Ω, 2.5 kV). The selection of *E. coli* clones was done on LB plates supplemented with 50 µg/ml kanamycin. The final constructs were verified by Sanger sequencing (Eurofins Genomics). Next, electrocompetent *C. koseri* SGSC4696 cells (wild-type and phage-resistant variants) were prepared. Briefly, a culture of 100 ml of mid-exponential *C. koseri* SGSC4696 cells was centrifuged four times (3000 × *g*, 15 min at 4 °C) and suspended in subsequent lower amounts of ice-cold 10% glycerol (40, 20, 10 and 1 ml). Cells were then transformed (25 μF, 200 Ω, 2.5 kV) with the recombinant plasmids (pET28a*::rfaF* and pET28a*::gt*) and selected using TSA plates supplemented with 50 µg/ml kanamycin.

### Bacterial challenge test

Early exponential phase (OD_620nm_ = 0.2) *Citrobacter* cultures (CK#1 and CK#2) were 10 × diluted to obtain ∼10^6^ CFU/ml and then challenged with CkP1 at MOI of 0.1 at 37 °C. SM buffer was used as a control. Next, the bacterial growth was monitored by turbidity (OD_620nm_) every hour. Complementary analysis was made to assess the phage antibacterial effect at 8 h of incubation, by plating serial dilutions of the suspensions with and without the CkP1 in 0.9% NaCl, and counting the CFUs after an overnight incubation at 37 °C. Averages ± standard deviations for all experiments are given for *n* = 3 repeats.

### Infection assays in urine

To complement the assessment of the antimicrobial potential of the phage, CkP1 was tested in artificially contaminated urine. Donors have signed their informed consent on collecting the urine samples. Mid-exponential growing CK#1 cells were washed once with TSB and then with filtered (0.22 µm) urine collected from healthy human subjects. Finally, cells were diluted in filtered urine to ~ 10^6^ CFU/ml. The contaminated urine samples (190 µl) were incubated with phages at a final multiplicity of infection of 0.1 (10 µl) or with SM buffer (10 µl). The suspension was incubated for 4 h and for 24 h at 37 °C. After, the cells were diluted in 0.9% NaCl, plated in triplicate, and CFUs were counted following an overnight incubation at 37 °C. Averages and standard deviations of four repeated experiments are given.

### Nucleotide sequence accession numbers

The complete genome sequence of the phage vB_CkoM_CkP1 was deposited in GenBank under the accession number MW239124. The sequencing reads of the *C. koseri* SGSC4696 variants isolated in this study were deposited in the European Nucleotide Archive (ENA, https://www.ebi.ac.uk/ena) under the study accession number PRJEB41322.

## Results

### CkP1 infects all *C. koseri *strains tested and with a high killing efficiency

Hospital clinical isolates of *C. koseri* were used to enrich wastewater treatment plant sewage samples, which resulted in the isolation of the phage vB_CkoM_CkP1 (CkP1) exhibiting uniform, clear, and small plaques (0.1 mm in diameter). A comprehensive panel of 60 bacterial strains was used to determine the phage host range and its efficiency of plating (Table 1). To our knowledge, CkP1 is the first described phage specific for *C. koseri*. Notably, CkP1 was able to infect all tested *C. koseri* strains from different origins, isolated from a 3-year period and which have distinct antibiotic resistance profiles. CkP1 also presented a high EOP on all *C. koseri* strains tested. In turn, CkP1 was unable to lyse closely related *Citrobacter* (*C. freundii*, *C. amalonaticus*, *C. youngae*, and *C. braakii*) and more distant *Enterobacteriaceae* species.

To further characterize the phage infection cycle, one-step growth curve experiments were established under standard growth conditions (Figure S1). CkP1 had a relatively short latent period of 25 min with an average burst size of 110 ± 38 PFUs per infected cell. Taken together, CkP1 seems to hold optimal characteristics for detection and/or treatment of *C. koseri* infections, including those caused by drug-resistant isolates.

### CkP1 features high stability, able to withstand various pH and temperatures

**Fig. 1:**
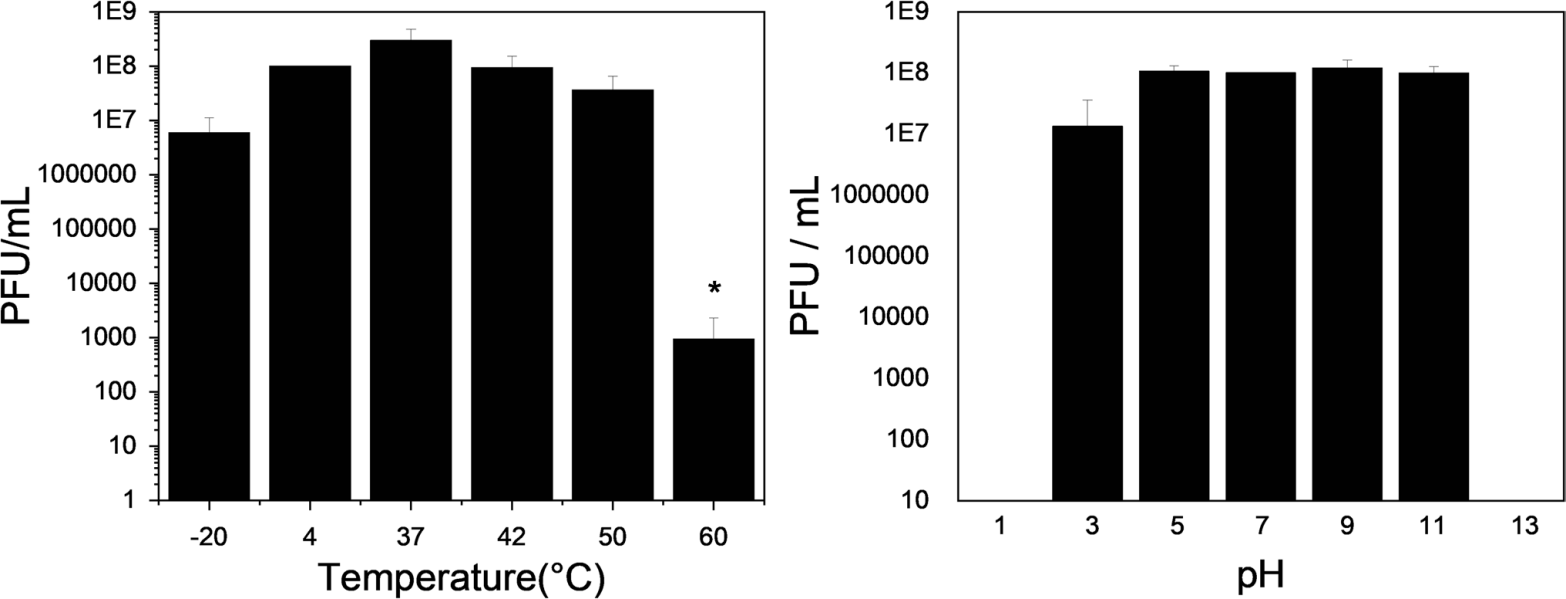
Phage CkP1 biophysical stability. Effect of different temperature (left) and pH (right) conditions on CkP1 phage survival after 24-h exposure by enumerating the number of phages. Averages and standard deviations of three repeated experiments are given. Significance was determined by Student *t* test (*P < 0.05)

To further assess this phage’s potential for therapy and detection, stability tests were set up, exposing CkP1 to different temperatures and pH values, for 24 h (Fig. 1). CkP1 remained stable between − 20 and 50 °C, maintaining its titer. At 60 °C, the CkP1 infection was significantly affected, with a titer decrease of 5 log units. Under different pH conditions, no loss of CkP1’s titer was observed after 24 h within the pH range of 5 to 11. At pH 3, CkP1’s titer reduced approximately 1 log unit and was completely abolished at extreme pH values of 1 and 13. Furthermore, CkP1’s titer remained unaffected for 2 years when stored at pH 7 and 4 °C (data not shown).

### CKP1 is a new S16-like myovirus

**Fig. 2:**
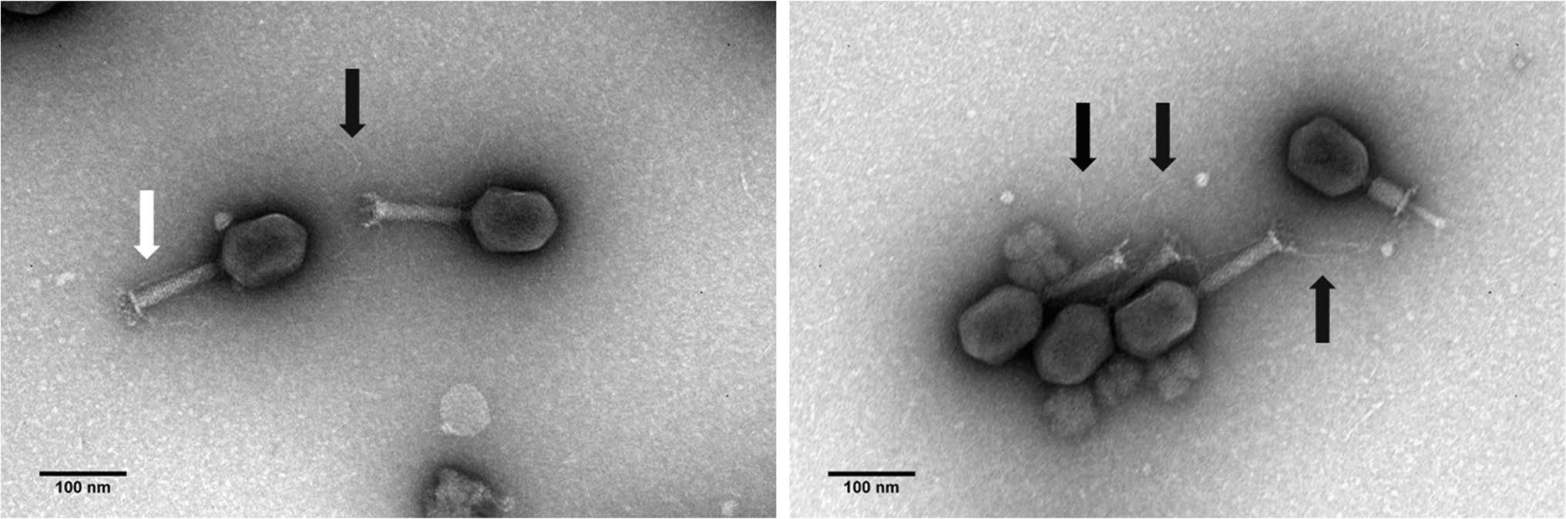
Phage CkP1 electron micrographs. Phage particles of CkP1 negatively stained with 2% uranyl acetate observed at TEM. Long tail fibers folded along the tail sheath (white arrow) or extended (black arrow) are indicated

Morphologically, TEM images showed that CkP1 has a myovirus morphotype (Fig. 2), composed of an icosahedral head with a contractile tail (head, 98 nm; tail, 133 by 18 nm) (Ackermann 1996). It was also possible to observe the different folding positions of the CkP1 tail fiber, stowed along the tail sheath and extended.

**Fig. 3:**
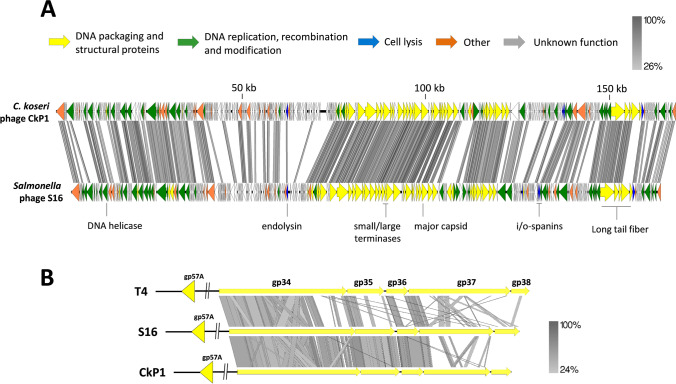
Phage CkP1 multiple genome alignment. **A** Whole-genome pairwise comparisons between the phage CkP1 (GenBank accession no. MW239124) and the *Salmonella* phage S16 (GenBank accession no. HQ331142). **B** Pairwise comparison of the long tail fiber region between the prototype *E. coli* phage T4 (GenBank accession no. NC_000866), *C. koseri* phage CkP1, and *Salmonella* phage S16. All comparisons were made using tBLASTX and visualized with EasyFig. CDSs are drawn to scale and colored according to their predicted function

Genomically, CkP1 has a linear DNA molecule with a size of 168,463 bp, encoding 291 putative genes and 11 tRNAs (Fig. 3A). The CkP1 GC content (36.9%) is similar to other *Citrobacter* phages (*e.g.*, Moon, Mijalis with GenBank accession no. KM236240, KY654690) or slightly lower (*e.g.*, CfP1, CVT22 GenBank accession no. KX245890, KP774835). CkP1 is related with the *Salmonella* phage S16 (Fig. 3A), a T-even broad host range phage that specifically recognizes *Salmonella* through OmpC (Marti et al. 2013). CkP1 shares 36% DNA overall homology and 62% of its proteins with those of phage S16, classified as a *Tevenvirinae* (T-even) of the *Straboviridae* family. The CkP1 genome structure resembles those found in T-even phages. The DNA packaging and structural module is tightly packed (gp165-211 and gp264-268), while the DNA replication, recombination, and modification and the cell lysis module, composed of the endolysin (gp131), a T type holin (gp269), and the o- and i-spanins (gp241-242), are scattered throughout the genome. Overall, CkP1 shares 38 out of 39 T4-like core-genes, *i.e.*, the most ancient genetic components of this group (Petrov et al. 2010). The prohead core scaffolding and protease is the only gene missing in CkP1. Still, one CkP1 orthologue is found in a similar genome position as of T4 genome and with similar function.

### CkP1 gp267 is a tail fiber able to decorate *C. koseri *cells

Based on in silico analysis, we identified the proximal to the distal parts of CkP1 LTF formed by gp264 and gp267 proteins, as well as their predicted accessory proteins involved in expression/trimerization (gp160/gp268). They are in perfect synteny with their LTF analogs (gp34-gp37 and gp57A/gp38) found in the type phages T4 and T2 in one hand and S16 on the other hand (Marti et al. 2013; Hyman and Raaij 2018) (Fig. 3B). While the proximal LTF parts of CkP1, *i.e.*, gp264-gp266 has relatively high similarity (> 60% average amino acid identity) to T-even proteins, the distal ends gp267 and gp268 exhibit moderate or low levels of identity (maximum of 54% and 36% amino acid identity to the homologs in T4 and T2, respectively). In addition, the structures of the N-terminal and C-terminal parts of gp267 were modeled using AlphaFold-Multimer. Both parts of gp267 are well predicted by AlphaFold, reaching global pLDDT scores of 86.0 and 87.6 for the N-terminal and C-terminal part, respectively (Figure S2). Notably, predicting the full gp267 structure results in a considerably lower pLDDT score (data not shown). Both FoldSeek and HHPred results indicate structural similarity between the C-terminal part of gp267 and the tip of the long tail fiber of T4 (gp37; PDB entry 2XGF; Table S1, Figure S3). This fragment also contains eight His-X-His sequences motifs (X being any amino acid), and its predicted structure forms a needle structure like T4 gp37 in which these histidine pairs coordinate iron ions (unlike gp37 of T2 or S16) (Bartual et al. 2010). Furthermore, neither FoldSeek nor HHPred show any meaningful structural similarity for the predicted N-terminal part of gp267 compared to other known structures (Figure S3). Note that the N-terminal parts of gp37 of T2 and T4 have not yet been crystallized, which could explain this finding. Overall, both predicted structures suggest that the gp267 of CkP1 LTF is a combination of a C-terminal part that is structurally similar to gp37 of T4 with an additional N-terminal part that is not structurally similar to any other known structure.

**Fig. 4:**
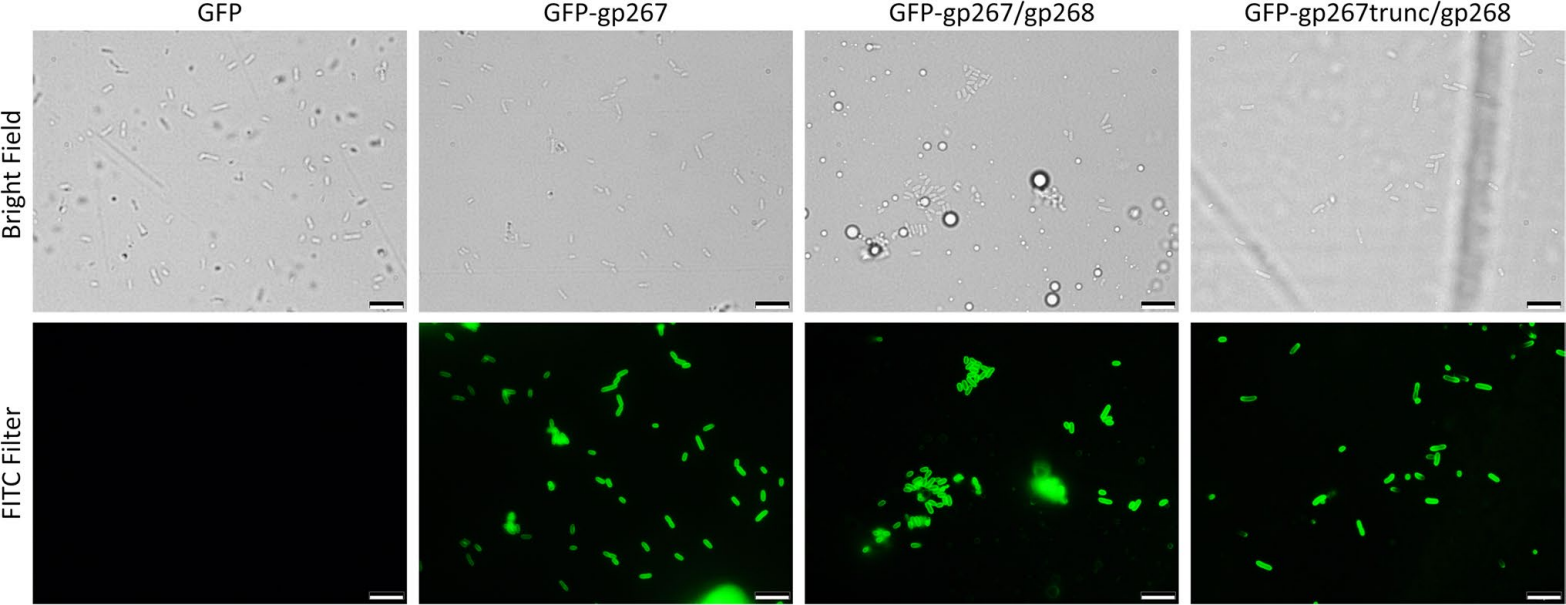
Phage CkP1 LFT binding of *C. koseri* cells. *C. koseri* cells (CK#1) suspended in 10 mM Tris–HCl (pH = 7) were incubated with GPF, GFP-gp267, GFP-gp267/gp268, or GFP-gp267trunc/gp268, washed twice and visualized under epifluorescence microscopy, in bright field or using the FITC filter

To functionally characterize the CkP1 LTF, we constructed three versions of gp267 fused to a GFP (GFP-LTF), which were co-expressed with and without the putative chaperone gp268 (referred to as GFP-gp267, GFP-gp267/gp268, and GFP-gp267trunc/gp268). The reasoning behind the gp267 LTF truncation variant was to isolate the putative receptor-binding sequence, which is located in the C-terminus. To assess the role of the gp267 LTF ligand, its potential binding activity was analyzed by epifluorescence microscopy. We observed that gp267 always bound to *C. koseri* cells independent of the construct (Fig. 4). Moreover, fluorescent microscopy experiments were performed using GFP-LTF proteins on all strains depicted in Table 1. All *C. koseri* strains were decorated by the GFP-LTF proteins while non-*koseri* strains from the *Citrobacter* genus or other closely related bacteria did not show fluorescent decoration on the microscopy experiments. This matched the GFP-LTF binding activity to the lytic spectrum of the phage CkP1, and resulted in a 100% specificity and sensitivity on the panel of strains tested.

### CKP1 LTF binds to cells with nanomolar affinity

The equilibrium binding affinities of the different CkP1 LTF versions against immobilized bacterial cells were analyzed by SPR. Kinetic data was obtained at different CkP1 LTF concentrations (1 nM to 1 µM) for each variant, and a global fitting of the data overall measured concentrations was performed. The best fit was obtained when using a predefined two-state reaction model, as previously described for the cell wall binding domain of the *Pseudomonas* phage endolysin KZ144 (Briers et al. 2009). It should be noted that this observation alone does not provide direct evidence for an actual two-state reaction, including a conformational change occurring during the binding of the CkP1 LTF to its cell wall receptor. This modeling enabled the calculation of association (*k*_a1_), dissociation (*k*_d1_), forward (*k*_a2_), and backward (*k*_d2_) rate constants, as well as the apparent equilibrium affinity constants (*K*), for all three variants of the LTF (Table 2, Figure S4). The *K* values obtained for GFP-gp267 and GFP-gp267/gp268 were 2.03 × 10^8^ M^−1^ and 8.41 × 10^7^ M^−1^, respectively, which corresponds to affinities in the low nanomolar range, whereas the equilibrium affinity constant for GFP-gp267trunc/gp268 was approximately tenfold lower (8.27 × 10^6^ M^−1^). Similar values were obtained when a “1:1 binding with mass transfer” model was applied for the fitting (data not shown).

**Table 2:** Kinetic data and equilibrium affinity constants (K) for the binding of CkP1 GFP–LTF variants to *C. koseri* Ck#1 cells. For each variant, a global fitting over six different concentrations (1 nM to 1 µM) was performed, using a “two-state reaction” model

LTF variant	k_a1_ (M^−1^ s^−1^)	k_d1_ (s^−1^)	k_a2_ (s^−1^)	k_d2_ (s^−1^)	K (M^−1^)
GFP-gp267	7.57 × 10^3^	9.65 × 10^**−**4^	4.80 × 10^**−**4^	1.93 × 10^**−**5^	2.03 × 10^8^
GFP-gp267/gp268	8.89 × 10^3^	2.02 × 10^**−**3^	5.27 × 10^**−**3^	2.90 × 10^**−**4^	8.41 × 10^7^
GFP-gp267trunc/gp268	1.22 × 10^3^	2.84 × 10^**−**3^	9.79 × 10^**−**3^	5.34 × 10^**−**4^	8.27 × 10^6^

### CkP1 recognizes *C. koseri* lipopolysaccharide through its LTF

To identify potential phage receptors, two different strategies were pursued. An initial approach (the Datsenko technique) used a direct knockout approach of potential gene candidates frequently involved in host recognition by T4-like phages. As a second approach, we mapped the missing genes of phage-resistant *C. koseri* variants that were sequenced and compared to the wild type genome (Table 3).

**Table 3:** Genetic variations in strains used to assess the CKP1 potential receptors. All variants used in this study derived from *C. koseri* SGSC4696 (GenBank accession n. NC_009792), being generated either using Datsenko technique (V1-V3) or by isolation of spontaneous mutants challenged with CkP1 (V4-V8), as described in the material and methods. In literature, the *rfaF* gene is also known as *waaF*. *SNV* single nucleotide variants. Below a summary of the spectrum of activity of both phage and its LTF against the different *C. koseri* variants generated in this study

Gene	Locus tag	Annotation function	Variant allele	V1	V2	V3	V4	V5	V6	V7	V8
*ompC*	CKO_RS02455	Outer membrane porin C	Indel: deletion	X	–	X	–	–	–	–	–
*ompA*	CKO_RS08945	Outer membrane porin A	Indel: deletion	–	X	X	–	–	–	–	–
*glgB*	CKO_RS20700	1,4-alpha-Glucan branching enzyme	SNV: G429 to E	–	–	–	X	X	X	X	–
*rfaF*	CKO_RS21630	ADP-heptose-LPS heptosyltransferase	Indel: frameshift insertion at pos. 9	–	–	–	X	–	X	X	–
*rfaF*	CKO_RS21630	ADP-heptose-LPS heptosyltransferase	SNV: L116 to R	–	–	–	–	X	–	–	–
*gt*	CKO_RS21655	Glycosyltransferase	Indel: frameshift deletion at pos. 78	–	–	–	–	–	–	–	X
Sensitive to phage CkP1				YES	YES	YES	NO	NO	NO	NO	NO
Decorated by GFP-LTF				YES	YES	YES	NO	NO	NO	NO	NO

In the first approach, *C. koseri* deletion mutants of OmpC (*ompC* at CKO_RS02455) and OmpA (*ompA* at CKO_RS08945) porins were created individually and combined, as they are often reported as receptors of T-even phages (Bertozzi Silva et al. 2016). However, none of the resulting mutants influenced phage infection, its EOP, or the targeted binding of GFP-LTF (Table 3).

As a second and complementary approach, five spontaneous resistant mutants to CkP1 were isolated. The GFP-LTFs were also not able to bind to these mutants, with the exception of variant V8 for which the cells were not completely decorated, but some green fluorescent dots were observed around the cells. The mutants were thus sequenced to identify potential mutations affecting the receptor. Genomic data demonstrated four small genetic variations as potential candidates for resistance-specific mutations located in the genes encoding the 1,4-alpha-glucan branching enzyme (*glgB*), ADP-heptose-LPS heptosyltransferase (*rfaF*), and glycosyltransferase (*gt*) (Table 3). While the role of the *glgB* is unclear, the *rfaF* and *gt* are predicted to be involved in the synthesis of the lipopolysaccharide. Two variations occurred in the *rfaF* gene encoding an ADP-heptose-LPS heptosyltransferase, which is involved in lipopolysaccharide biosynthesis (KEGG ortholog K02843). A frameshift insertion was found at codon 9 in three mutants and another mutant carried a substitution of leucine to arginine at codon 116. All four mutants carrying an *rfaF* variant also carried a glycine to glutamic acid substitution in another gene, *glgB*, which encodes an 1,4-alpha-glucan branching enzyme involved in starch and glucose metabolism (K00700). The fifth mutant was affected by a frameshift deletion in the *gt* gene. Interestingly, the mutated gene is located in close vicinity (~ 4,400 bp) of *rfaF*. No additional larger genomic variations (insertions, deletions, or rearrangements) were found in the mutant strains. In an attempt to validate the phenotypic effect of these mutations, we performed *in-trans* complementation of *rfaF* and *gt* genes, but not for *glgB* gene, which failed to be amplified by PCR. Nevertheless, we observed that the complemented mutants were again infected by phage with high EOP. Accordingly, the CkP1 GFP-LTF protein versions could also decorate all the complemented mutants (Figure S5).

### CkP1 can inhibit Citrobacter growth

To complement the assessment of the phage CkP1 antimicrobial properties, we challenged two *C. koseri* strains (CK#1, CK#2) with the phage at MOI of 0.1 and monitored the OD over time (Figure S6). In the absence of CkP1, the cultures grew exponentially to an OD value of approximately 1.0. In the presence of CkP1, both cultures were inhibited of growth until at least 8 h. At this incubation period (8 h), CkP1 reduced 4 logs of both CK#1 and CK#2 cultures, comparatively with cells without phage treatments. Therefore, CkP1 proved to be efficient to reducing *C. koseri* in rich media.

### *C. koseri* cells are efficiently controlled by CkP1 in urine samples

**Fig. 5:**
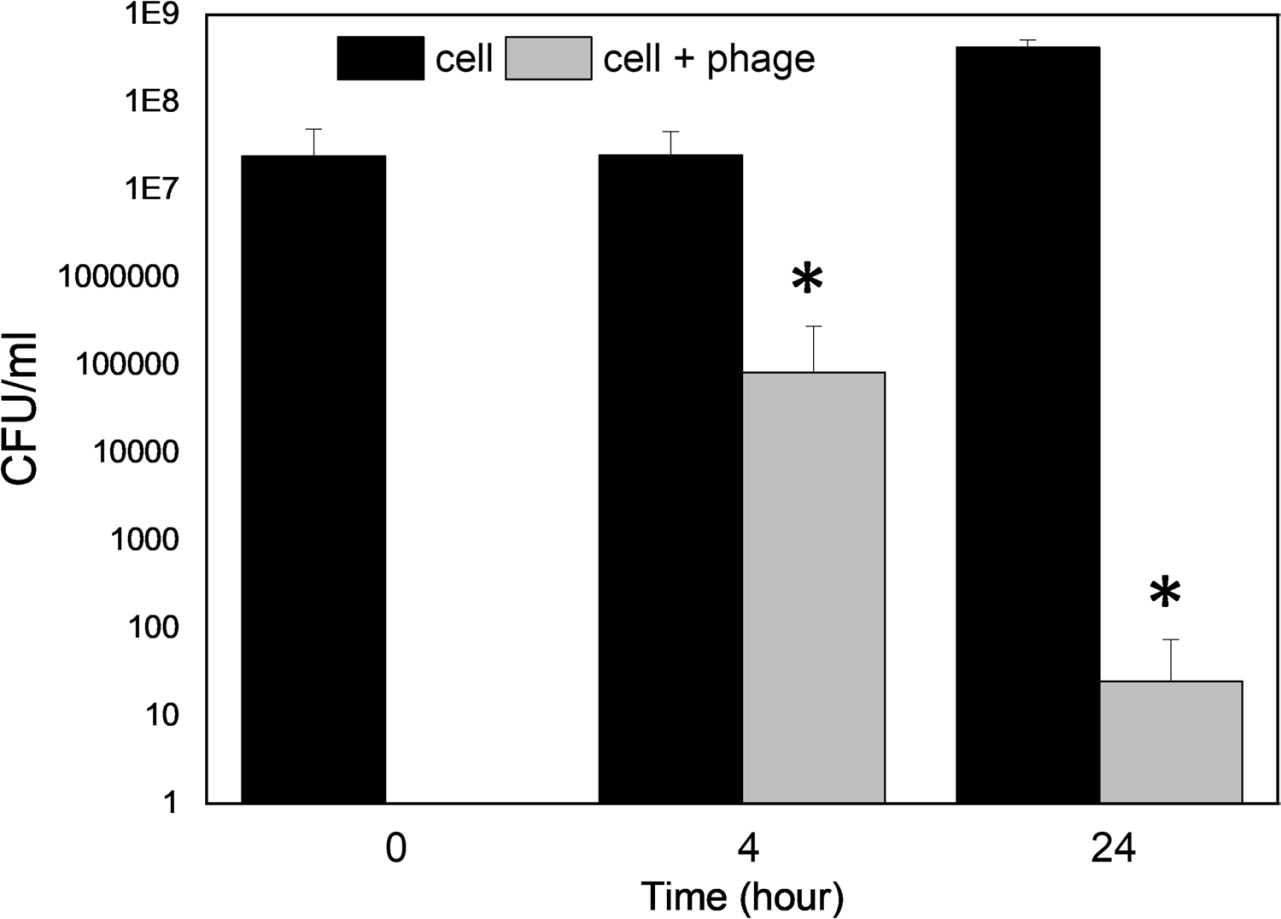
Control of *C. koseri* in urine with CkP1. Antibacterial effect of CkP1 on urine samples artificially contained with *C. koseri* (CK#1). Phage was applied at a MOI of 0.1 and the antibacterial effect measured by reduction of bacterial CFUs/ml at 4 h and 24 h. Significance was determined by the Student *t* test (*P < 0.05). Averages and standard deviations of four repeated experiments are given

To mimic treatments for urinary tract infections, the antimicrobial effect of CkP1 was assessed in an in vitro setting, using urine samples collected from healthy donors (Fig. 5). These samples were mixed with mid-exponential phase *C. koseri* cells and further incubated with CkP1 at a MOI of 0.1. In the absence of phages, *C. koseri* cells were able to grow 1 log in cell number after 24 h incubation at 37 °C. In the presence of phages, CkP1 bacterial counts were reduced by proximally 2 logs and 5 logs (6 logs if we consider the control at the same time point) after 4 h and 24 h of incubation, respectively. It can therefore be concluded that CkP1 treatment can efficiently control *C. koseri* cells in spiked urine under these relevant conditions.

## Discussion

*Citrobacter* spp. are ubiquitous Gram-negative, facultative anaerobic bacteria that belong to the *Enterobacteriaceae* family. Currently divided into 8 members, *C. koseri* (formerly named *C. diversus*) is among the most common isolated species in human clinical specimens able to cause localized (urinary track, wounds infection, pneumonia, meningitis) and systemic life-threatening diseases (bacteremia, septicemia). Urinary tract infections are often associated with drug-resistant *C. koseri* (Ranjan and Ranjan 2013) and therefore alternative treatments are needed.

In this study, we isolated and characterized a novel T-even phage, CKP1, specific for *C. koseri*. CkP1 shares 38 out of 39 T4-like core genes (Petrov et al. 2010), but contains one orthologue possibly covering the missing prohead core scaffolding and protease gene function. Whole-genome comparisons place the broad host range *Salmonella* phage S16 as the closest CkP1 homolog. Similarly, CkP1 also infects all strains but limited to the *C. koseri* species, not infecting other closely related species. The *C. koseri* strains were obtained from public collections and clinical specimens isolated from different sources, time points (spanning three years) and which display distinct antibiotic-resistance patterns.

Our initial efforts attempted to identify if common outer membrane proteins previously described in T-even phages (porins OmpA and OmpC) (Bertozzi Silva et al. 2016) were also the receptors of CkP1. After single gene knockouts and double null mutants of these porins (*ompC* at CKO_RS02455 and *ompA* at CKO_RS08945 locus), the resulting strains remained sensitive to the phage and could still be infected with high efficiency. As an alternative approach, we selected spontaneous bacterial variants that survived phage predation, and were able to pinpoint indels and SNVs in three genes (*glgB*, *rfaF*, and *gt*). *rfaF* and *gt* genes are located within the *C. koseri* SGSC4696 rfa (also known as waa) operon (4,705,399–4,716.689 bp) predicted to be involved in the synthesis and assembly of the LPS, similarly to *E. coli* rfa operon (Pagnout et al. 2019). Mutations on *rfaF* (also known as *waaF*) have been shown to severely truncate the lipooligosaccharide composed of Kdo and lipid A (Kanipes et al. 2006), whereas the lack of the glycotransferase affects the distribution of sugar moieties (Leipold et al. 2007). As for the branching enzyme *glgB* gene involved in the glycogen biosynthesis pathway, it is found outside the *rfa* operon, so it is unclear whether its function is related to LPS biosynthesis. Four out of the five *C. koseri* variants studied contained mutations in *glgB* together with *rfaF* or *gt* genes. Due to unknown reasons, we were not able to amplify the *glgB* coding sequence by PCR and subsequently to complement the mutant *glgB* allele. Therefore, we can currently not fully interpret the role of *glgB* within this context at this time. The strict co-occurrence of the allele variant of *glgB* with variations in *rfaF* hints towards a connection between these genes, but phage infection of the successful complementation of *rfaF* variants proved that the *glgB* allele is not essential for resistance against CkP1. CkP1 infectivity of the defective phenotypes could be fully restored by *in-trans* complementation with *rfaF* or *gt* genes from the wild-type host, supporting that phage CkP1 recognizes *C. koseri* cells through the lipopolysaccharide polymer, possibly binding to the inner core.

Here we also describe a novel T-even LTF that functions without the need of any accessory proteins. The T-even (*e.g.*, T4, T2, Bp7, and S16) LTF model is well described and composed of gene products gp34 to gp37 that form the proximal to distal segments (King and Laemmli 1971, Dunne et al. 2018; Chen et al. 2020; Salem et al. 2021). However, in the case of T4, the functional LTF (gp37) requires additional molecular chaperones (gp57A and gp38) (Hashemolhosseini et al. 1996). For T2 and S16, the LTF (gp37) needs to be co-expressed with the gp38 that functions as an adhesin, attaching to the C-terminal part of the mature LTF and modulating the receptor specificity (Riede et al. 1985, 1987; Marti et al. 2013). Here we proved that the expressed proteins of CkP1 gp267 LTF or a truncated C-terminal part (homolog of the T-even gp37) were able to decorate *C. koseri* cells. However, the role of gp268 remains unclear (orthologue of the T-even gp38 nomenclature), which apparently does not need to be co-expressed with the gp267 LTF to obtain a functionally active LTF able to decorate cells under epifluorescence microscopy. It also seems unlikely that gp267 contains a C-terminal chaperone, as it appears to form a needle structure just like the C-terminal end of gp37 of T4 LTF. These results were further validated by SPR following a protocol established to measure interactions of phage-derived proteins with the bacterial cell walls (Loessner et al. 2002; Schmelcher et al. 2010; Marti et al. 2013). In this assay, the reported apparent equilibrium affinity constants represent a measure for the accumulated binding strength between the possibly multimeric protein and the cell surface, taking into account possible avidity effects. For instance, duplication of the cell wall binding domain (CBD) of a phage endolysin resulted in an approximately 50-fold increase in equilibrium association constant (KA) as compared to the parental single-CBD protein. This could be explained by the simultaneous binding of up to two cell wall ligands by the dual-CBD construct (Schmelcher et al. 2011). In our experiments, we showed that there were no differences in the observed nanomolar range affinity equilibrium between the LTF (gp267) co-expressed with or without the additional protein (gp268). The CkP1 LTF binding kinetics were also equivalent to those observed in other described T-even LTF that need accessory proteins (Marti et al. 2013). Moreover, CkP1 LTF was able to decorate all phage-sensitive *C. koseri* strains used in this study, but not the phage-resistant *C. koseri* mutants or non-host strains, matching its binding to the phage lytic spectrum. *In-trans* complementation of these mutants again allowed cell decoration by the GFP-LTF, confirming that the lack of phage infectivity of the mutants is related to the phage adsorption/recognition through its LTF (gp267). Of note, mutation of the *gt* gene did not completely abolish binding of gp267 either because the resulting modification on the LPS is not substantial to hinder binding or gp267 binds only partially to the LPS affected by the *gt* mutation. The high affinity of this phage LTF, coupled with the demonstrated 100% specificity and sensitivity for *C. koseri*, shows the high potential of gp267 as a biorecognition element in the development of new diagnostic tools to specifically detect this pathogenic bacterium.

Additional tests show that the phage CkP1 has a strong antibacterial activity against *C. koseri*, widening the repertoire of characterized *Citrobacter*-infecting phages previously limited to *C. freundii* (*e.g.*, CfP1, phiCFP-1, SH1-SH5) (Hamdi et al. 2016; Oliveira et al. 2016a, b; Zhao et al. 2016) and *C. rodentium* (CrRp3 and CrRp10) (Mizuno et al. 2020) species. In comparison to other *Citrobacter* myoviruses (CfP1 and CrRp10), CkP1 features similar infection parameters but superior stability towards extreme environmental conditions, comparable with *C. freundii* phage CfP1, previously isolated by our group (Oliveira et al. 2016a, b). While both withstand temperatures ranging from − 20 to 50 °C and pH from 3 to 11, CkP1 is also able to tolerate to some extent, temperatures of 60 °C after a 24-incubation period. Furthermore, we demonstrate that CkP1 does not quickly induce resistant phenotypes and that can control *C. koseri*-driven urinary tract infections. This high stability and the host range covering the whole species make CkP1 phage a promising candidate to specifically control and/or detect *C. koseri* pathogens, including drug-resistant isolates.

## Supplementary Information

Below is the link to the electronic supplementary material.

Supplementary file1 (PDF 844 KB)

## Data Availability

All data generated or analyzed during this study are included in this published article and its supplementary information files.
