# Supplementary material for: CkP1 bacteriophage, a S16-like myovirus that recognizes Citrobacter koseri lipopolysaccharide through its long tail fibers
Source: Appl Microbiol Biotechnol. 2023 May 3;107(11):3621–36. doi: 10.1007/s00253-023-12547-8 (PMC10175313; doi:10.1007/s00253-023-12547-8)
Supplement: Supplementary file 1 — Supplementary file1 (PDF 844 KB) [file 253_2023_12547_MOESM1_ESM.pdf]

Supplementary material

Journal Name: Applied Microbiology and Biotechnology

Article Title: CkP1 bacteriophage, a S16-like myovirus that recognizes Citrobacter koseri lipopolysaccharide through its long tail fibers

Author Names and Affiliations: Hugo Oliveira[1,2*], Sílvio Santos[1,2], Diana P. Pires[1,2], Dimitri Boeckaerts[3], Graça Pinto[1,2], Rita Domingues[1,2], Jennifer Otero[1,4], Yves Briers[4], Rob Lavigne[5], Mathias Schmelcher[6], Andreas Dötsch[7] and Joana Azeredo[1,2*]

[1] Centre of Biological Engineering, University of Minho, Braga, Portugal (hugooliveira@ceb.uminho.pt, silviosantos@ceb.uminho.pt, priscilapires@deb.uminho.pt, gracapinto@ceb.uminho.pt, ritadomingues1898@gmail.com, jenniferoterocarrera@gmail.com, jazeredo@deb.uminho.pt)

[2] LABBELS –Associate Laboratory, Braga, Guimarães, Portugal

[3] Department of Biotechnology, Ghent University, Ghent, Belgium (dimtri.boeckaerts@ugent.be; yves.briers@ugent.be)

[4] Departament de Genètica i de Microbiologia, Universitat Autònoma de Barcelona, Barcelona, Spain (jenniferoterocarrera@gmail.com)

[5] Department of Biosystems, KU Leuven, Leuven, Belgium (rob.lavigne@kuleuven.be)

[6] Institute of Food, Nutrition and Health, ETH Zurich, Zurich, Switzerland (mathias.schmelcher@hest.ethz.ch)

[7] Max Rubner-Institute, Institute for Physiologie and Biochemistry of Nutrition, Karlsruhe, Germany (andreas.doetsch@mri.bund.de)

[*] Corresponding author:

Hugo Oliveira (hugooliveira@deb.uminho.pt)

Joana Azeredo (jazeredo@deb.uminho.pt)

Tel. + 351 253 604 419 Fax. + 351 253 604 429

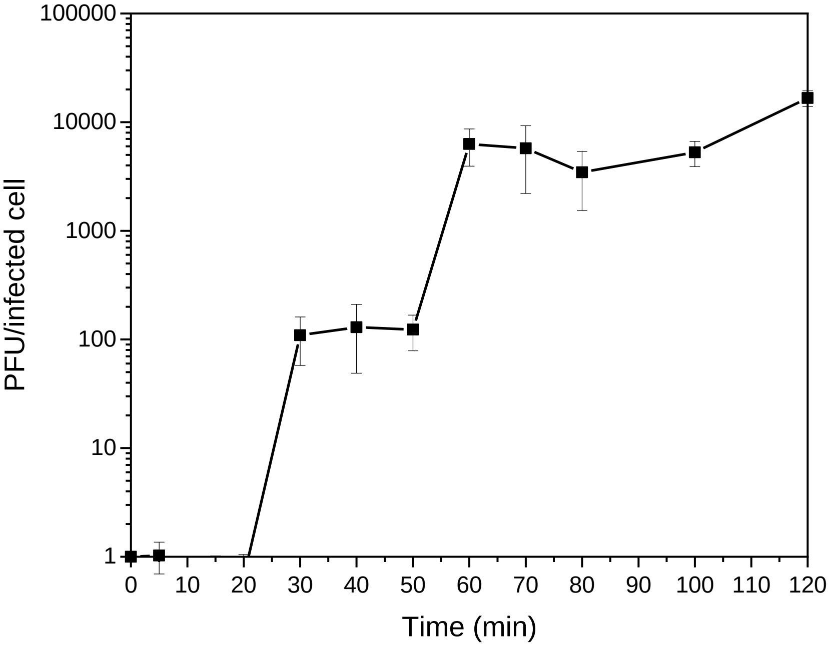

Figure S1. Phage CkP1 one-step growth curve. Phage experiment was performed using a culture of *C. Koseri* CK#1. Data present mean ± standard deviation from three independent experiments.

a) Sequence coverage and pLDDT scores of the predicted structures for the N-terminal part of gp267.

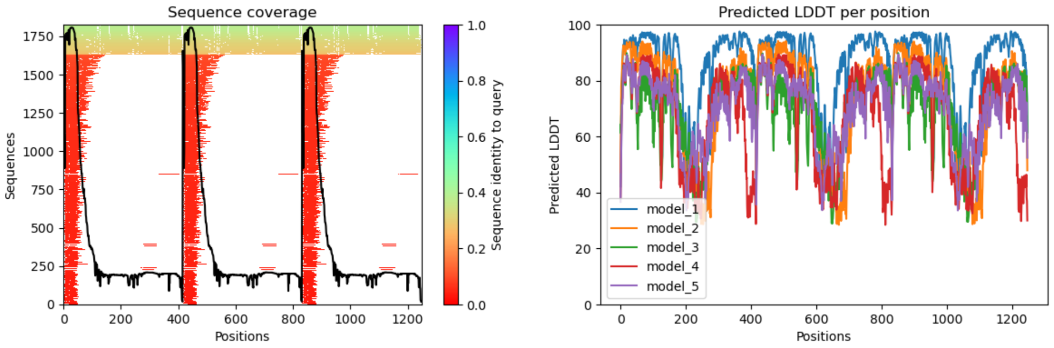

b) Sequence coverage and pLDDT scores of the predicted structures for the C-terminal part of gp267.

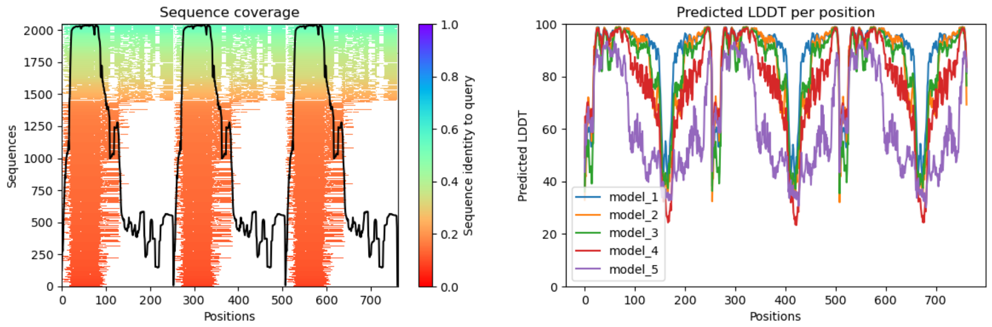

Figure S2: Sequence coverage and pLDDT scores for each of the predicted structures of (a) the N-terminal and (b) the C-terminal part of CkP1 gp267 (visualized with ColabFold).

Table S1.1. Structural similarity search of the N-terminal part of CkP1 gp267 with HHPred and FoldSeek. These results show no significant structural similarity to other known structures.

Method	Best hit	Probability	Score	E-value	Target length
HHPred	2C3F	37.5%	33.48	82	358
FoldSeek	4YHC	/	153	0.0827	411

Table S1.2. Structural similarity of the C-terminal part of CkP1 gp267 with the T4 gp37 (PDB entry: 2XGF).

Method	Probability	Score	E-value	Target length
HHPred	97%	/	0.013	242
FoldSeek	/	376	4.521e-7	213

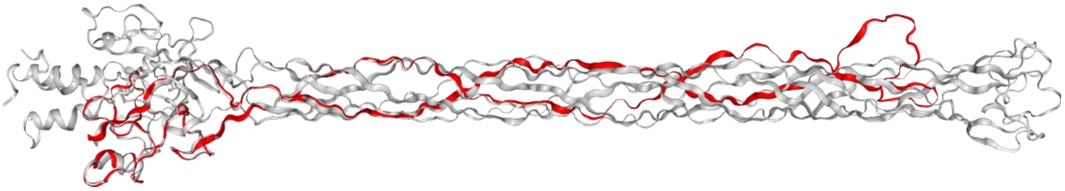

Figure S3.1. FoldSeek. FoldSeek visualization of the overall structural similarity between the predicted structure of the C-terminal part of CkP1 gp267 (in grey) and T4 gp37 (in red, here only visualized as a monomer).

a) **Predicted structure of the N-terminal part of CkP1 gp267**

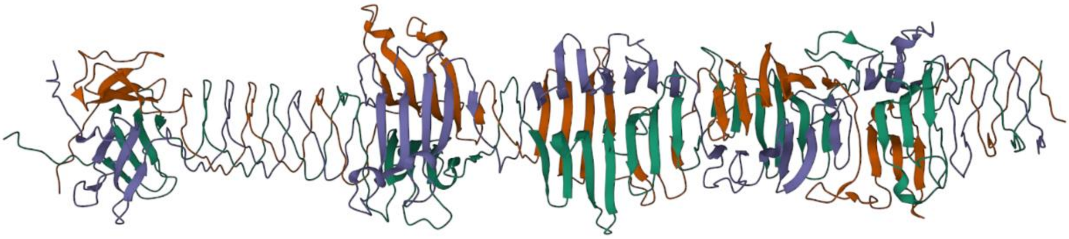

a) **Predicted structure of the C-terminal part of CkP1 gp267**

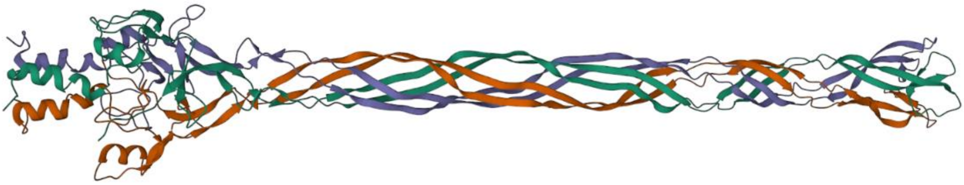

Figure S3.2. AlphaFold-Multimer. Predicted structures of the N-terminal part and C-terminus of gp267 with AlphaFold-Multimer. The (highest-scoring of five) predicted trimeric structures of both (a) the N-terminal part of gp267 (global pLDDT of 86.0, pTM of 74.6%) and (b) the C-terminal part of gp267 (global pLDDT of 87.6, pTM of 74.0%) indicate that the C-terminal part of gp267 forms a needle structure like the long tail fiber from T4 (gp37), while the N-terminal part predominantly forms parallel β-sheets.

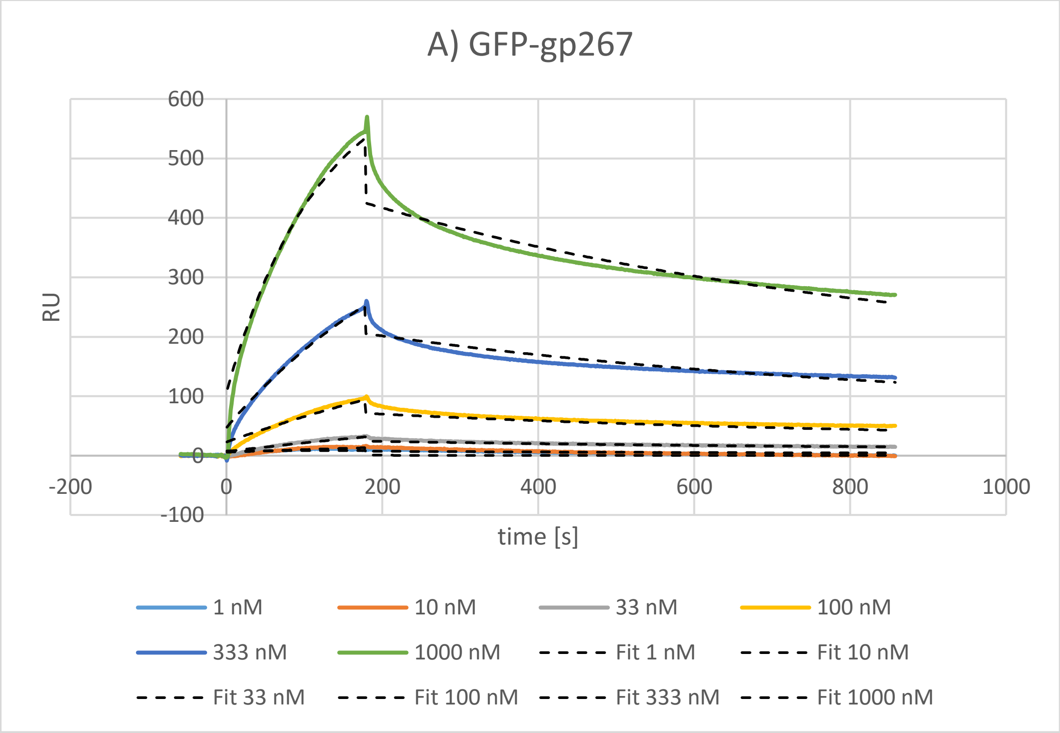
A) GFP-gp267
RU
600
500
400
300
200
100
0
-100
-200
0
200
400
600
800
1000
time [s]
1 nM
10 nM
33 nM
100 nM
333 nM
1000 nM
Fit 1 nM
Fit 10 nM
Fit 33 nM
Fit 100 nM
Fit 333 nM
Fit 1000 nM

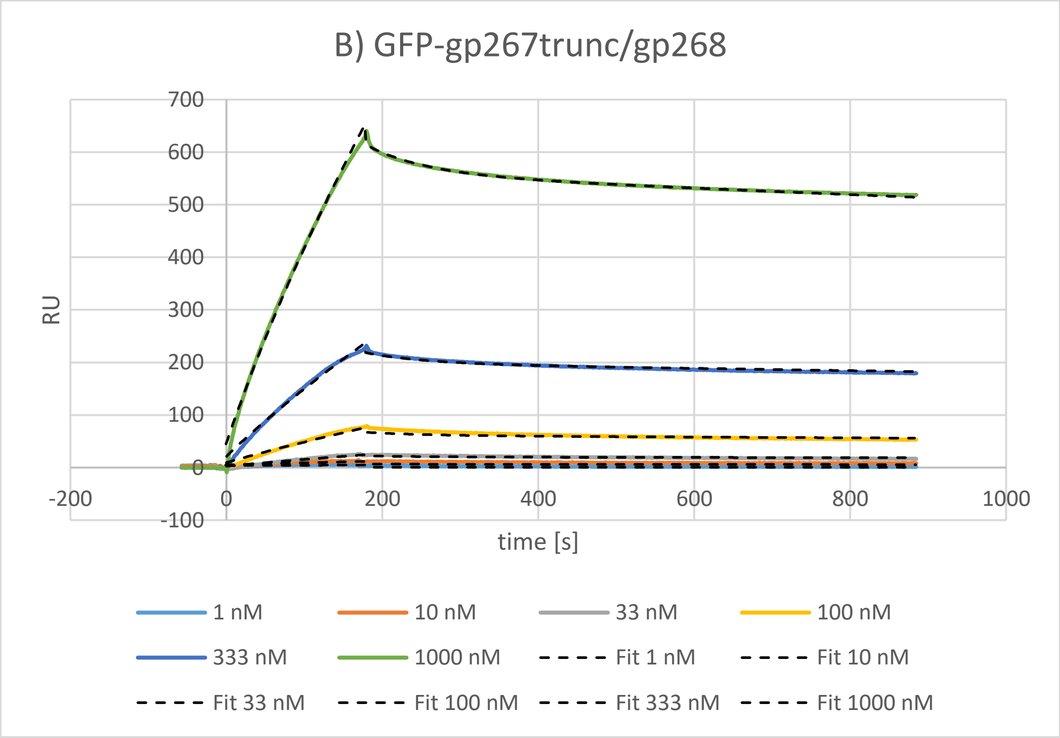
B) GFP-gp267trunc/gp268
RU
700
600
500
400
300
200
100
0
-100
-200
0
200
400
600
800
1000
time [s]
1 nM
10 nM
33 nM
100 nM
333 nM
1000 nM
Fit 1 nM
Fit 10 nM
Fit 33 nM
Fit 100 nM
Fit 333 nM
Fit 1000 nM

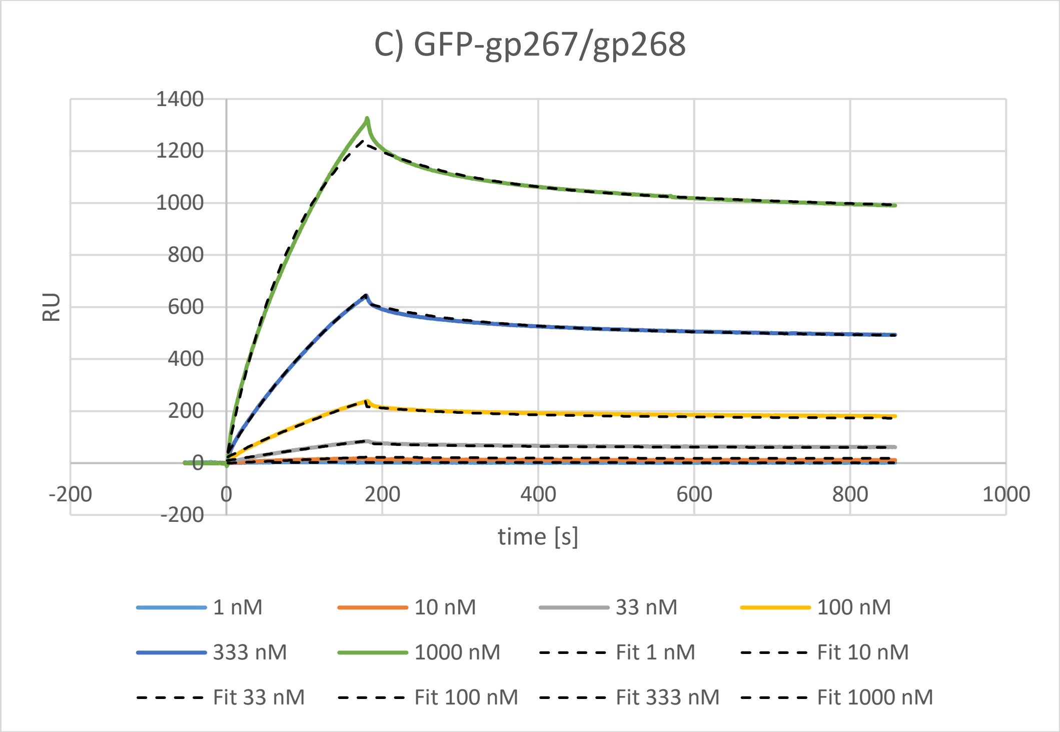

Figure S4. SPR analysis of tail fiber proteins binding to the surface of *Citrobacter* cells. SPR sensorgrams showing real-time interactions of GFP-gp267 (A), GFP-gp267trunc/gp268 (B), and GFP-gp267/gp268 (C) with the cell surface of *Citrobacter koseri* (Ck#1), measured at different analyte concentrations. Association phases were recorded for 180 s and dissociation phases for 720 s. Dashed lines represent fitting of the data to a "two-state reaction" model, using a global fitting approach. RU, relative response units.

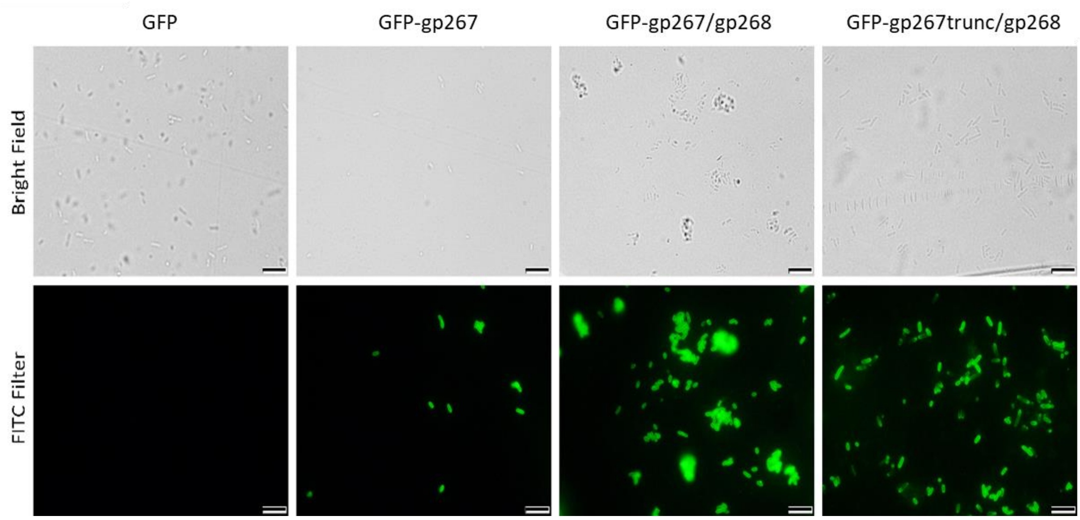

Figure S5. Phage CkP1 LFT binding to complemented resistant mutant of *C. koseri*. *C. koseri* cells of a complemented resistant mutant (V7 complemented) suspended in 10 mM Tris-HCl (pH = 7) were incubated with GPF, GFP-gp267, GFP-gp267/gp268 or GFP-gp267trunc/gp268, washed twice and visualized under epifluorescence microscopy, in bright field or using the FITC filter.

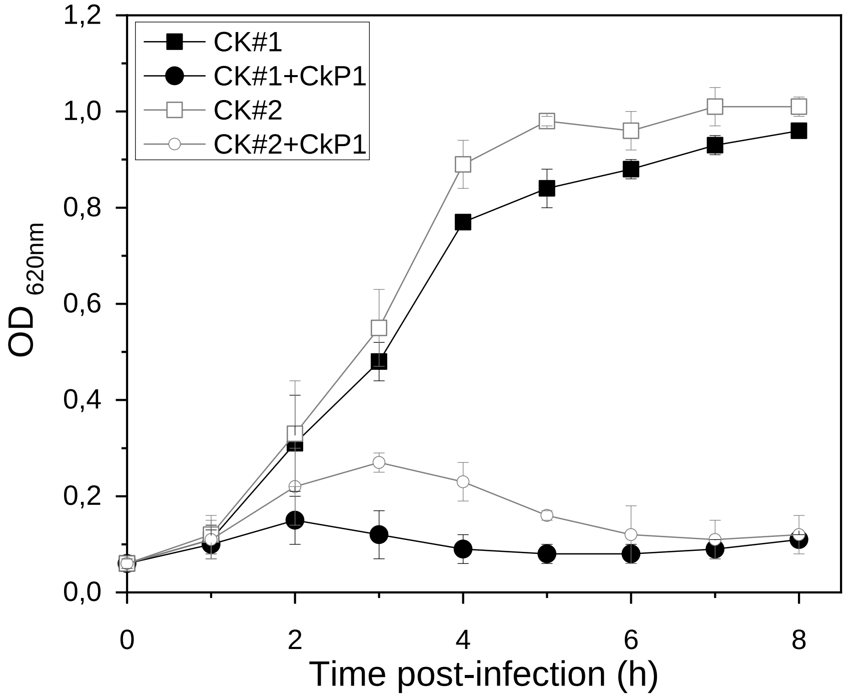

Figure S6. ***C. koseri*** **challenge tests.** Early exponential phase *C. koseri* cultures (CK#1 and CK#2) at ~10^6 CFU/ml were challenged with phage CkP1 at MOI of 0.1 at 37ºC. SM buffer as used as a control. Turbidity (OD_{620nm}) was measured overtime.
